# The meniscus-guided deposition of semiconducting polymers

**DOI:** 10.1038/s41467-018-02833-9

**Published:** 2018-02-07

**Authors:** Xiaodan Gu, Leo Shaw, Kevin Gu, Michael F. Toney, Zhenan Bao

**Affiliations:** 1 Department of Chemical Engineering, Stanford University, Stanford, CA 94305 USA; 2 Stanford Synchrotron Radiation Lightsource, SLAC National Accelerator Laboratory, Menlo Park, CA 94025 USA; 3 Present Address: School of Polymer Science and Engineering, University of Southern Mississippi, Hattiesburg, MS 39406 USA

## Abstract

The electronic devices that play a vital role in our daily life are primarily based on silicon and are thus rigid, opaque, and relatively heavy. However, new electronics relying on polymer semiconductors are opening up new application spaces like stretchable and self-healing sensors and devices, and these can facilitate the integration of such devices into our homes, our clothing, and even our bodies. While there has been tremendous interest in such technologies, the widespread adoption of these organic electronics requires low-cost manufacturing techniques. Fortunately, the realization of organic electronics can take inspiration from a technology developed since the beginning of the Common Era: printing. This review addresses the critical issues and considerations in the printing methods for organic electronics, outlines the fundamental fluid mechanics, polymer physics, and deposition parameters involved in the fabrication process, and provides future research directions for the next generation of printed polymer electronics.

## Abstract (web summary)

A primary advantage of polymer semiconductors compared to silicon-based semiconductors lies in its capability of being solution-processed for the large-scale fabrication of electronics that can be flexible, stretchable, implantable, biodegradable, and self-healing. Here, Gu and Shaw et al. review recent developments in meniscus-guided coating that can control thin-film morphology.

## Introduction

One of the primary advantages of organic semiconductor (OSC) devices is their ability to be fabricated with solution-phase processing methods onto flexible substrates. In contrast to most silicon-based devices, which typically require high-temperature melt processing or chemical vapor deposition, OSC devices are amenable to high-throughput, large-area deposition like roll-to-roll (R2R) printing processes, which facilitate lower-energy and more versatile fabrication. Due to their unique electronic properties and intrinsic mechanical flexibility, semiconducting polymers have seen widespread use in devices ranging from photovoltaics (OPVs)^1^ and field-effect transistors (OFETs)^2^ to emerging technologies such as electronic skin^3^, stretchable electronics^4,5^, and photodetectors^6^, thereby expanding application spaces^7^ and opening new ones underserved by conventional silicon technologies.

Small-molecule OSCs have traditionally led the field in terms of high-performance devices, but high-mobility polymers are becoming increasingly competitive^2,8^. Both have exceeded the performance of amorphous silicon and can even possess charge carrier mobilities within the same order of magnitude as polycrystalline silicon^9^. In addition to advances in the optimization of intrinsic material properties, a crucial consideration for the development of high-performance devices lies in the manner by which they are processed^10^.

A wide range of solution-based methods have been employed for the deposition of polymer semiconductor layers, with spin coating as the most commonly used technique in research-scale devices. While spin coating has an advantage in small-scale fabrication because of its simplicity and availability, the technique is wasteful of materials and is a batch process with limited throughput and industrial relevance. In addition, spin coating does not produce a uniform shear stress distribution during coating and may make controlling film morphology difficult. Mounting evidence in the literature demonstrates the crucial influence of deposition method on film morphology and draws attention to the importance of finely tuning processing parameters^10–13^. In particular, precise control over polymer thin-film morphology, which not only includes the crystalline domains for semicrystalline materials (e.g., degree of crystallinity, grain size, and grain orientation)^14^ but also the amorphous domains (e.g., tie chain density, chain orientation, and alignment)^11,15^, is important for good charge transport properties^2^ (a short description appears in section Controlling morphology and device performance).

Meniscus-guided coating (MGC) techniques are well-poised to address these concerns. The term “meniscus-guided” refers to the fact that a meniscus is translated across a substrate by virtue of a coating head or viscous forces, in effect guiding and controlling film deposition. Common MGC methods include dip coating, blade coating, solution shearing, and slot die coating^10^. Because of the intrinsic directionality of the coating process, MGC techniques can confer molecular alignment of the deposited OSC layer^16^ and are quite amenable to continuous, steady-state printing, such as in R2R processing. Unlike spin coating, where ~ 90% of the material is discarded, material utilization in MGC can be upwards of 99% using R2R processes in large-scale applications.

The performance and solution processability of semiconducting polymers is highly dependent on material and processing parameters. In polymer semiconductor solutions, one of the key parameters is appropriate solvent selection. Poor solubility may lead to unfavorable aggregation, time-dependent viscosity changes, and potential gelation, but the common use of halogenated solvents (which are generally good solvents) in lab-scale studies poses a significant environmental hazard for industrial-scale processes, potentially limiting manufacturing. Designing appropriate polymer structures that yield high mobility while retaining solution processability and favorable behavior during morphology evolution is a delicate process that has spurred on vigorous research aimed at turning this art into science^17^.

This review is a survey of the deposition of polymeric semiconductor thin films using MGC techniques and the materials and processing-related parameters that need to be addressed for the fabrication of high-performance devices with desired functionality. More importantly, we aim to provide insights and a critical review of research on MGC deposition and recently reported techniques used to control printed thin-film morphology. We further highlight areas requiring additional scientific inquiry. While many other researchers have published important contributions to the field, we regret that length constraints require their omission from this review.

## Solution processing

### Fluid mechanical phenomena

On the laboratory scale, small-scale deposition processes that are facile for demonstrating charge transport characteristics for newly synthesized materials are often used to provide proofs of concept. However, in order to scale up, a firm understanding of the fundamental fluid mechanical processes is needed, and the many factors that influence the resulting flow and shear fields must be accounted for in order to rationally control the deposition process. Toward this end, we describe below the relevant considerations common for MGC methods and emphasize that such issues should guide future research.

Box 1 summarizes the variety of fluid flows and gradients that affect polymer nucleation, aggregation, and alignment in solution at the primary meniscus. For most MGC methods, there is contact between the solution and part of the coating apparatus (such as a die nozzle or coating blade) so that the meniscus is the liquid–air interface that connects the coating head to the substrate, where the solution, substrate, and air form a three-phase contact line. Before solution enters the region under the influence of the meniscus, the velocity field established by the coating head can itself alter the morphology of the resulting thin film (vide infra). For simple blade coating with 0° tilt angle (blade parallel to the substrate), the system resembles classical parallel-plate Couette flow, and the Navier–Stokes equation can be solved exactly to yield a linear velocity profile. When the gap distance between the two plates is very small, the Reynolds number—Re = *ρνL*/*μ*, where Re relates the fluid density *ρ*, the relative velocity of the parallel plates *ν*, the gap distance *L*, and the fluid viscosity *μ*—becomes very small and reflects the diminished influence of inertial forces compared to viscous forces. Stokes flow refers to these low Re situations and is governed by a set of linearized (and thus approximate) steady-state Navier–Stokes equations, which allow for analytical solutions to more complex flows when Re « 1.

In the general blade coating case (non-zero tilt angles) when the gap height between the coating head is very small compared to the lateral dimension along the coating direction, the scenario becomes the classical slider-block (or “slider-bearing”) problem, and the Reynolds equations of lubrication theory can be used to approximate the velocity profile—which consists of a parabolic component caused by the pressure gradient (Hagen–Poiseuille flow) and a linear component (Couette flow)—and to estimate the shear strain up until solution enters the forward meniscus. For modest tilt angles, the shear field is only modestly affected by the pressure term in the equations, which intuitively originates from the passage of a volume of incompressible liquid from a larger cross-sectional area through the small gap before entering the meniscus. The shear strain is instead primarily boundary-driven, dominated mostly by the motion of the coating head (or equivalently, the substrate). Such a mathematical treatment of the slider-block geometry applies only for Newtonian fluids, which is an assumption valid for relatively dilute polymer solutions. More concentrated solutions or ones with very high-molecular-weight polymer may exhibit non-Newtonian (shear rate-dependent) behaviors like shear thinning, shear thickening, or even (time-dependent) thixotropy, which may have deep implications for control of thin-film morphology. As we will see, the region before the meniscus under the coating head is where control of the fluid flow can dramatically influence the resulting film.

One problem with these approaches, however, is that different flows are very likely induced by the process of thin-film formation downstream close to the drying front. A priori, we know that a dramatic concentration gradient is established within the meniscus—the concentration of solute increases from the initial value of the solution entering the meniscus to the bulk density of the dry solute in the film. As the polymer concentration rises near the contact line, the viscosity of the solution also begins to diverge as the material solidifies and is no longer a liquid. Because solvent evaporation is enhanced closer to the contact line as opposed to closer to the coating head, a capillary flow toward the contact line—also known as the coffee-ring effect^18,19^—can be induced. Depending on the solvent, it is possible that a surface tension gradient also arises along the meniscus, which can cause Marangoni flow^20^ to either recirculate solution back toward the coating head (in the case of surface tension gradients arising from differential evaporation in solvent mixtures) or enhance the flow toward the contact line (in the case of solutal Marangoni flow^21^). Moreover, because the meniscus functions as the evaporation front for the solvent, a temperature gradient exists and can cause complex flow patterns because the Marangoni effect and the temperature gradient can cause flows in opposite directions^22,23^. The relative importance of each of these phenomena leads to complicated relationships among each of these factors^24^.

Lastly, in the evaporative regime (explained further in the next section), the movement of the contact line is directly influenced by the rate at and manner by which the dissolved solute is deposited. Stick-and-slip behavior is quite common and often manifests as undulations in dry film thickness. A competition between a surface tension-related pinning force and a depinning force related to bulk fluid motion^25^, stick-and-slip phenomena result from the time-dependent processes involved with mass transport to the contact line, the material’s solidification from solution, and solvent evaporation^26^. The meniscus is elongated during the “stick” phase as the substrate continues to move with respect to the bulk fluid until it rapidly contracts back to a shortened length, leaving behind a thick, stripe-like solute deposit where the meniscus was pinned. When other fluid dynamical processes like viscous fingering (convective concentration instabilities) or density-dependent Rayleigh–Bénard convection are pronounced, the deposits can be shaped like dots or more elaborate structures^27,28^. This periodic distortion of the meniscus is a function of solution concentration, solvent evaporation flux, solute convection, shear viscosity, coating speed, height of the wetting film, and substrate surface energy^21,25,29–31^. While much work has been conducted in the dynamics and statics of wetting^32,33^, little of this knowledge has been directly applied to polymer OSC device fabrication beyond simple substrate surface treatments, which are often used to enhance crystalline ordering with only a subsidiary purpose of enhancing solution wetting for thin-film deposition.

It is clear that the ultimate morphology and (semi-)crystalline microstructure of polymer thin films deposited by MGC methods results from the concerted action of each of these processes. While additives, appropriate solvent selection, solvent blending, substrate surface treatments, etc. can be used to individually tune the relative importance of a given phenomenon, their combined effect is difficult to predict. Currently, coupled simulations incorporating all of these processes is computationally infeasible given the complexity of modeling solvent evaporation with solute crystallization and precipitation. Although attempts have been made to individually model portions of the system with simplifying assumptions^21,34^, a combined simulation that unifies flows induced within the meniscus with the velocity profile upstream and the contact line would allow for more effective modeling of the film deposition process.

### Deposition regimes

Box 2 summarizes the two fluid mechanical deposition regimes characterized by the resulting dry thin-film thickness: one in which film thickness first decreases with coating speed (or web speed) and one in which film thickness increases^35,36^. The thickness of the wet film is a function of the capillary number Ca = *µν*/*γ*_d_, which gives a measure of the relative effects of viscous forces over surface tension. Here *µ* is the solution viscosity, *ν* is the relative velocity between the web and blade, and *γ*_d_ is the surface tension of the liquid in ambient atmosphere. For low deposition speeds, solvent evaporation from the meniscus occurs on a timescale comparable to that of solid film formation, resulting in a film-drying process that is under the direct influence of the meniscus, the fluid flow fields within, and any shear forces imparted. This “evaporative regime”—known also as convective assembly—is characterized by a power-law decrease in dry film thickness as a function of increasing coating speed (*t* ∝ *v*^−1^) due to a shorter deposition time per unit length^37^. It is in this regime that stick-slip phenomenon is often found, and this cyclic process results in periodic variation in film thickness that may be undesirable for devices requiring highly uniform films. Furthermore, in multicomponent solutions, differential solute diffusivities may result in the spatial heterogeneity of each of the components in the final film when solidification happens over these relatively larger timescales.

As web speed is increased, the transition region occurs where film thickness begins to increase with coating speed into the classical Landau–Levich (LL) regime^37^. At high print speeds, viscous effects dominate, and a wet film is first dragged out before solvent evaporation deposits the resulting film. Unlike in the evaporative regime, the back meniscus (upstream of the primary, forward meniscus; Box 2a) plays an important role in film deposition by acting as the origin of pressure^35^. By first dragging out a wet film, the effects of the coating mechanism are effectively decoupled from the solid film formation because of the relaxation time for polymer chains (or aggregates) to change conformation (or orientation) is fast compared to solvent evaporation. Still, practical considerations include avoiding long evaporation times (i.e., minutes or more) and dewetting of the film from the substrate, which can cause the pooling of solution from the edges toward the center and lead to lateral nonuniformity via the coffee-ring effect previously mentioned^18^.

The transition between the two regimes occurs at a coating speed determined by a variety of factors. Increasing the solution concentration, deposition temperature, and aspiration rate of the surrounding inert carrier gas (i.e., air or nitrogen) directly enhance solvent evaporation, shifting upward the thickness curve as a function of coating speed in the evaporation regime. However, it is unclear whether or not the transition into the LL regime is moved toward higher speeds, but decreased solution viscosity and (possibly) increased polymer-solvent diffusivity can push this transition point toward the higher coating speeds^34^. Depending on the application, the feasible operating window of coating speeds within either the evaporative or the LL regimes can be adjusted by tuning these parameters. The typical transition speed is around a few mm s^–1^ for dilute conjugated polymer solutions at room temperature in a solvent with a boiling point near 125 °C.

### Solution formulation

While a more complete understanding of the complex interplay among fluid mechanical phenomena is important, another element of the deposition process that has received less attention is solution formulation. Although the requirements for properties such as fluid viscosity are less strict than for techniques like inkjet printing, such factors can have a variety of effects on the resulting films. For example, adjustment of solution viscosity and surface tension to manipulate film thickness often comes at the expense of solvent choice or concentration optimization for the particular OSC application. Very importantly, the environmental friendliness of the chosen solvent is crucial to the industrial-scale deployment of solution-processed OSC devices^38,39^. It is believed that the success of OPVs is dependent on scale-up by factors of millions or more; in order to truly realize global production capacity, non-toxic and non-halogenated solvents must be used, further restricting the allowable parameter space. In another example, polymer concentration has a straightforward effect on film thickness, increasing the coating-speed-dependence of the dry film thickness. However, the effect of concentration on semiconducting polymer aggregation, nucleation, and crystal growth—as well as the possibility of non-Newtonian behavior and other fluid mechanical considerations (see section Fluid mechanical phenomena)—during MGC is less clear and requires further study.

Recent trends in the study of organic photovoltaics have revealed the importance of solvent selection in the microstructural evolution of polymer thin films. For most bulk heterojunction (BHJ) OPVs, the solution is at least a ternary-component system consisting of electron-donor molecules, acceptor molecules, and solvent. Reports have shown that the use of binary solvent blends consisting of a high- and a low-boiling-point solvent allows for more controlled evolution of the final microstructure because of the former’s lower evaporation rate^40–42^. It is believed that this allows for residual solvent molecules to plasticize the polymer by virtue of remaining in the film for a longer period of time^43^. However, this vision of solvent evaporation posits boiling point as one of the most important parameters for solvent selection and blending and ignores deviations in total vapor pressure, an approximation of evaporative flux, caused by each of the components of the solution. Generally, the binary solvent mixture is composed of two species whose chemical dissimilarities would engender activity coefficients that are not unity^44^. Net associative interactions among the molecules of the two solvents would cause a negative deviation from Raoult’s law, decreasing the expected vapor pressure and indicating that more solvent remains in the drying film than anticipated. Such a scenario does not necessarily require solvents with differing boiling points and can also partly explain why recent OPV work has converged to using small amounts of additives like 1,8-diiodooctane to enhance film morphology. However, for these quaternary-component systems, each set of pairwise interactions may have important effects on how solvated the polymer components are, which can affect polymer–polymer interactions in solution, the polymers’ hydrodynamic shape during MGC, nucleation of a given species during drying, and the process of phase separation as the film evolves into its final microstructure. Such interactions may reveal alternative explanations—other than plasticization—for the improved film morphology induced by solvent mixtures and are in need of further inquiry.

Whereas some work has begun to address polymer–polymer interactions through the lens of Flory–Huggins solution theory by looking at their interaction parameter *χ*^45^, a complete analysis requires the consideration of all components of the system and their specific pairwise interactions. Even for simpler binary systems consisting only of polymer and solvent, solvent choice is often overlooked and if it is not, attention is only paid to the solvent’s boiling point to explain good or poor film morphology. Since semiconducting polymers are chemically heterogeneous amalgams of conjugated cores (sometimes with very different alternating mers) and insulating side chains, the two major associative interactions—alkyl stacking to form lamellae and *π*-stacking—may be modulated to differing degrees by a given solvent. In this way, Hansen solubility parameters resolve the simple Hildebrand solubility parameter into three constituent values each corresponding to a different type of associative interaction and would facilitate a more complete understanding of solvation power for rational solvent selection^46^. The equilibrium solution-phase aggregate (if aggregating) or free polymer coil (if not) likely differs in size and shape if the solvent quality is changed and may affect^47^ or be affected differently by the velocity and shear fields induced during MGC. Here solution-phase X-ray or neutron scattering can play an important role in quantitatively describing polymer chain configuration^48^. Furthermore, because aggregates likely serve as the nuclei for crystallite formation in semicrystalline polymers, solvent choice plays a pivotal role in controlling thin-film microstructure and reinforces the fact that proper ink formulation informed with a firm understanding of the fluid dynamical relationships among the deposition method, the polymer, and the solvent is needed for the rational control of solid film formation.

### Printing techniques

Printing techniques can be broadly classified into contact and non-contact methods, differentiated by whether a surface—e.g., the printing head, etc.—comes in direct, physical contact with the substrate to be coated. Contact methods include gravure printing, screen printing, and flexographic printing, which to date have not been commonly utilized in OSC processing. Conversely, non-contact methods include blade coating, slot die coating, and inkjet printing; meniscus-guided methods are a subset of non-contact methods. Here we describe several MGC techniques to give the reader a brief overview of the MGC landscape, and in particular, we highlight slot die coating due to its industrial relevance.

Among the simplest MGC technique is dip coating, in which a substrate is vertically withdrawn from a solution reservoir. Depending on the withdrawal speed, solute concentration, solution viscosity, and reservoir temperature, either a dried (evaporative regime) or liquid thin film (LL regime) can be deposited. It should be noted that dip coating may be done with a single substrate (i.e., immersion and subsequent withdrawal) or in a continuous R2R configuration.

In contrast, a variety of similar techniques in which a solid edge is passed over a bead or reservoir of solution can be classified under as “blade coating.” Typically, blade coating is described as translating a sharp blade held perpendicular to the substrate over a solution droplet. The forward meniscus is formed at the trailing edge, and the ink reservoir upstream of the meniscus is exposed to the ambient. A cylindrical or rectangular bar may be also used, which is often referred to as “bar coating.” Solution shearing is a similar technique where an angled blade is used with a temperature-controlled substrate, which induces a controlled flow profile on the solution and also effectively restricts evaporation of the rear upstream meniscus. From a fluid mechanical perspective, blade coating is effectively horizontal dip coating, meaning the same evaporation and LL regimes are present. However, in dip coating the primary source of shear strain is viscous rather than boundary-driven, as in the case when a solid blade is used. Blade coating is commonly encountered in the literature as it is a lab-scale analog to industrial-scale coating methods^49^.

### Slot die coating

Slot die coating is extremely high throughput and has a large tolerance for ink viscosity ranging from 10^–3^ to 10^3^ Pa⋅s. Because of this, slot die coating is a prominent MGC method for industrial applications^50^ and warrants discussion in the context of polymer semiconductors. Slot die coating involves a hollow die head through which solution is pumped onto a moving web at a fixed substrate-to-head (gap) height. Capillary forces hold the liquid bead between the web and die head as the web is translated at a fixed speed. One of the main advantages of slot die coating is that it is a pre-metered, continuous process—specification of web width, speed, and ink flow rate (from a controlled pump) determines the resulting thin film thickness, within stable operating parameters (Box 2b). At slow speeds, solvent evaporation occurs at the downstream forward meniscus, resulting in a solid film deposited at the air–liquid–solid interface. Such slow evaporative-regime slot die coating is commonly referred to as zone casting. For semiconducting polymer solutions, this typically entails slow speeds on the order of tens of microns per second. Understandably, for industrial-scale manufacturing it is generally more attractive to operate in the LL regime to attain high throughput (often at speeds greater than meters per minute). Colloquially, and for the purposes of this review, the LL regime is implied in the term “slot die coating.” In this case, the slot die head achieves steady-state coating equal to the liquid feed rate. Controlling process variables enables the deposition of a uniform wet film that dries homogenously between a few and several hundred microns thick.

The limits of achievable wet film thickness are acutely dependent on a range of variables, including but not limited to solution viscosity, surface tension, web speed, pump rate, and die head geometry (Box 2b)^51^. A key concern is determining the window of operating limits in such a vast parameter space^52^. Improper operating conditions may lead to defects and nonuniform film thickness, if any solid film is deposited at all. Analytical solutions of the governing fluid dynamical equations exist for very few idealized situations but can provide important parameters like the minimum wet film thickness *t*_min_ and the zero-pressure-difference film thickness *t*_0_^50^. The first theoretical study of slot die coating operating limits was presented in 1976^53^, but despite a wealth of research over several decades, a comprehensive unified understanding of the underlying mechanisms remains elusive. It is fair to say that the overarching theme of understanding slot die coating stability is that it is presently—for all practical purposes—largely empirical. Even numerical methods by solving the 2-D Navier–Stokes system of equations struggle to determine limits of instability^50^.

For *Ca* « 1, capillary forces dominate fluid flow, holding the fluid between the gap and enabling stable coating flow. At high Ca, capillary forces are unable to prevent viscous forces from disrupting the flow profile as the liquid film is dragged out. However, it should come as no surprise that flow stability cannot be simply reduced to a single dimensionless quantity. There exist analytical models for stability windows albeit with varying degrees of fluid ideality—namely, inviscid^53^, viscous^54^, and viscocapillary flow; we direct the reader to others’ work for a detailed mathematical treatment^50^. While such models can relate geometric and operating parameters to *Ca*, they are usually restricted to specific conditions resulting from their underlying assumptions—e.g., small Ca and small Re. It should be noted that these analyses are also presented by assuming Newtonian fluid behavior. Since slot die coating has been used for fabricating organic thin films relatively recently, there have not been many detailed studies of the fluid mechanical properties of the OSC solutions themselves. Polymer solutions may exhibit shear-thinning or viscoelastic behavior, which are important considerations given that fluid elasticity generally acts to destabilize the downstream forward meniscus during coating.

Naturally, web speed is limited to some maximum value, beyond which specific failure modes occur. The downstream meniscus is generally pinned to the lip of the slot die head in stable coating flow, but the upstream rear meniscus may fail to pin to the back lip of the die head under low-flow or high web speed conditions. In between the uniform homogenous film and completely unstable coating regions lies a defect region above a critical Ca. Here dynamic wetting failure can lead to defects such as ribbing, air entrainment, and rivulets (Box 2b). Furthermore, even within the stable coating window, vortices may also be formed, causing defects or nonuniformities in the printed film. The subset of conditions constituting vortex-free flow is often a small fraction of the stable coating window. In general, the most important factor in determining vortex-free conditions is the lip geometry and angle, but again, vortex avoidance is largely empirically determined.

## Controlling morphology and device performance

The performance of organic electronic devices is closely related to the morphology of the deposited thin film, specifically to the way that the molecules pack in the solid state^15^. Films of conjugated polymers are especially complex because of both their semicrystalline nature and the extensive disorder within their crystalline domains compared to that of small-molecule crystalline domains. In this section, we will discuss the recent use of MGC to enhance OSC device performance by addressing general phenomena that affect charge transport as it pertains to polymer OFETs and OPVs. For the reader’s convenience, we have compiled key device parameters and data for the references cited in this review in Supplementary Data 1. It is worth noting that these two devices involve very different ideal film morphologies. Single-material devices, OFETs, involve charge transport that occurs in-plane with the substrate near the dielectric–organic interface. The primary figure of merit for OFET performance is the charge carrier mobility, which is typically measured directly from thin-film transistor devices. The measured charge carrier mobility from transistors is a combination of intra- and inter-chain transport, given that the transistor channel length is on the order of tens of microns, a length scale that is significantly larger than contour length of a single polymer chain with a molecular weight <100 kDa. Promoting the charge transport along the polymer backbone by either designing a coplanar polymer backbone to enhance electron delocalization^55^ or synthesizing high-molecular-weight molecules greatly benefits the device performance^56^. Moreover, a higher degree of crystallinity in conjugated polymers together with tie chains can assist the charge hopping process for inter-chain charge transport;^57^ further discussion on OFET charge transport can found in the reviews by others^58–60^. In OPVs, the phase-separated domain size between the two semiconducting materials and efficient out-of-plane charge transport within those percolated domains to the electrodes are understood to be two of the most important device morphology considerations. It is clear that film morphology can dramatically affect device performance, and rational control of the deposition processes is crucial. We refer further discussion of morphology–property relationships for OPVs to other reviews^1,12,61^.

### Nucleation control

The ordered crystalline domains in semicrystalline conjugated polymer thin films play an important role in charge transport. Polymers with high crystallinity are desirable for use in high-performance OFET devices, although a recent result has indicated that high crystallinity is not always a prerequisite for effective charge transport^55,57,62^. Nonetheless, because crystalline order can directly affect device performance, understanding the nucleation and crystal growth behavior of these materials is needed. Precise control of these two processes for polymer films deposited by MGC is not easy compared to crystalline, small-molecule systems. While theoretical and experimental work on homogeneous and heterogeneous nucleation in non-conjugated polymer systems is extensive, less effort has been focused on semiconducting polymers. However, while detailed studies of the nucleation of conducting polymers are scarce, insights to approach the problem of conducting polymer nucleation and crystallite growth can be gleaned from work on conventional insulating polymers like semicrystalline polyethylene (PE), which has been studied extensively to determine the mechanism underlying crystallization from the melt and solution. We briefly discuss such mechanisms and refer the reader to other authorities on polymer crystallization for further reading^63^.

The crystallization of a polymer from the melt results in the substantial decrease in conformational entropy of the polymer chain and can be separated into three sequential regimes: initial formation of nuclei; nucleus extension and coalescence; and finally crystal growth (Box 3)^64–67^. As compared to small molecules, the key difference in the polymer nucleation process is the ability for a single chain to be incorporated into more than one nucleus (for sufficiently high molecular weights). The associative interactions between polymer segments result in the formation of aggregates because of either undercooling below the lamella “equilibrium melting temperature” *T*_m_° or supersaturation caused by solvent evaporation. At *T*_m_°, the aggregates exist at the critical lamella thickness L* beyond which crystal growth becomes thermodynamically favorable. This size is given by L* = 2*σ*_e_*T*_m_°/(Δ*H*_f_ Δ*T*) + *δ*, where *σ*_e_ is the surface energy of the fold, and Δ*H*_f_ is the enthalpy of fusion at *T*_m_°. The undercooling is given by Δ*T* = *T*_m_° − *T*, and *δ* is an empirical correction to account for a minimum L* when Δ*T* is extrapolated to infinity. After aggregates are formed but before they reach L* in size, these nuclei, which may include polymer chains that connect multiple nuclei together, either dissociate or grow by incorporating new polymer chains or unincorporated chain segments attached to other nuclei. Both mechanisms of enlargement involve, to some degree, translation and adjustment of the backbones into energetically favorable conformations in the growing nucleus^68^, and nucleation is complete when the lamella achieves a thickness L* after a maturation time t*. The crystallization from solution is analogous to that from melt, as the driving force now changes from cooling to supersaturation of the polymer solution during solvent evaporation.

The lamellae of PE crystals consist of polymer backbone “stems” incorporated perpendicular to the plane of the lamella, which can dynamically rearrange during nucleation and growth. These PE polymers have molecular weight that are typically on the order of 10^6^ Da; have short persistence lengths (~ 0.7 nm)^69^ and are therefore flexible; and are simple homopolymers without complicated side chains or moieties. Because it is predicated on such model polymer molecules, polymer nucleation theory as developed by Hoffman, Weeks, and Lauritzen is most applicable to such classes of materials. In contrast, donor–acceptor conjugated polymers have relatively rigid backbones that reduce main chain torsion and that make the polymers stiff or rod-like^69^. Typical molecular weights range from 10^4^ to 10^5^ Da, making these polymers shorter than conventional ones and possibly reducing the density (and influence) of inter-chain entanglements. The primary intermolecular forces are *π* interactions in addition to associative dispersion forces among the alkyl side chains. These forces are considerable for crystallizable conjugated polymers, often leading to a deviation of crystallization behavior from that of simple polyolefins because the free energy landscape for polymer conformational rearrangements, nucleation, and crystallization is significantly different from that of simple polymers like PE (Box 3e). Such interaction often results in the presence of solution-phase aggregates and/or liquid crystalline (LC) behavior, which add another layer of complexity in understanding the crystallization process for conjugated polymers^48^. Nonetheless, estimations of parameters like Δ*H*_f_ or *T*_m_°^70^ may prove useful for future conjugated polymer design, and although the heterogeneous nature of the polymers may entail the development of more complicated theoretical paradigms, the insights developed in the past 60 years are not entirely upended and provide a firm foundation to build new frameworks for the study of conjugated polymers.

**Fig. 1:**
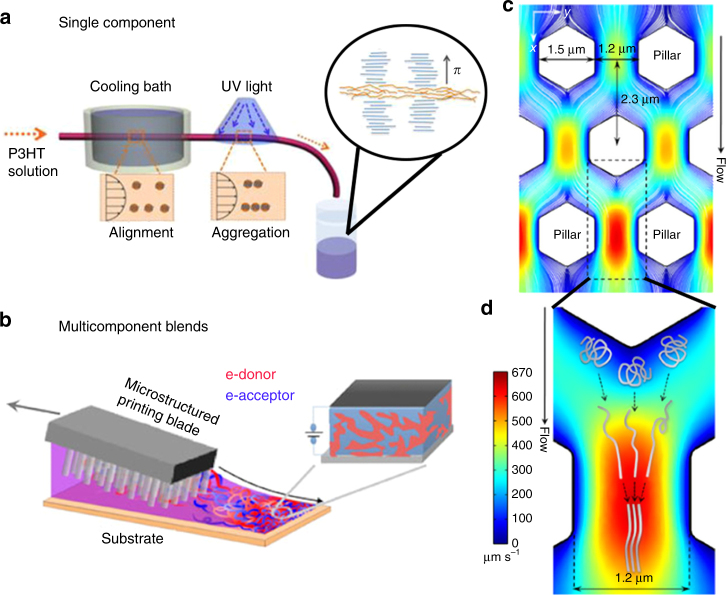
Nucleation induced by deposition. **a** Pretreatment of polymer solutions under shear flow can nucleate pseudo-stable P3HT aggregates from polymer molecules (red dots) in solution, which can subsequently be cast. The aggregates are “shish-kebab” structures with *π*-stacked polymer molecules (blue) attached along the long axis (orange) of the aggregate core. Figure adapted from ref. ^91^ (copyright 2015 American Chemical Society). **b** Enhancement of the nucleation of a polymer component in the matrix of an amorphous, co-deposited polymer can result from the use of patterned coating blades that locally increase shear strain and introduce extensional flow. Fluid dynamical simulations of the pillar array (**c**) indicate variations in fluid velocity (denoted with color) that both contribute to higher shear strain and cause extensional flow (**d**) as fluid upstream of two adjacent pillars converges between them. Although this method is shown for a binary-component mixture of two polymers, the principle is likely general for semicrystalline polymers. Figure adapted from ref. ^79^ (copyright 2015 Nature Publishing Group)

Of more practical interest to the MGC of these materials are the parameters that can be tuned to control their nucleation during deposition. As mentioned before, ink formulation plays a crucial role in the development of the final film morphology and can be optimized. For example, nucleation agents have been commonly used to alter polymer morphology and overall crystallinity^71^ and, when used for semiconducting polymers, have been shown to increase the crystallization rate of poly(3-alkylthiophene-2,5-diyl) (P3AT)^72^. Furthermore, solid “crystallizable” solvents have been used to nucleate (and align^73^) poly(3-hexylthiophene-2,5-diyl) (P3HT) with the presence of unidirectional temperature gradients^74^. However, an additional nucleation additive is sometimes unneeded: in conventional polymers, it has been found that metastable aggregates remain in solution when the bulk material is quickly dissolved and then later act as nuclei for further crystallization. This “self-seeding”^75^ has also been exploited in conjugated polymers—solution-phase aggregation processes can be manipulated prior to deposition by aging, sonication, shear flow (Fig. 1a), or a combination thereof^76^. In many of these cases, the resulting nucleation density and film morphology is highly dependent on the exact chemical nature of the polymer OSC and the additive, if present, making predictions challenging.

Shear-induced crystallization is a phenomenon that can also be exploited to control polymer crystallization during MGC. The use of shear forces during a solution-phase processing technique is a general method to induce nucleation supported by a mature body of literature for conventional polymers^77,78^. There are a few examples of the application of such techniques on polymer OSCs in OPV devices. Patterned coating blades can manipulate the fluid dynamics of solution shearing during the deposition of all-polymer solar cells (Fig. 1b) and enhance their performance^79^. By increasing extensional flow and the overall shear strain imparted on the solution, shear-induced nucleation of the donor polymer was enhanced, as demonstrated by the increase in the relative degree of crystallinity that increased solar cell performance—in particular, the overall power conversion efficiency (PCE) improved by nearly twofold.

Lastly, heterogeneous polymer nucleation onto various substrates is another example that for conventional polymers has been studied extensively^80,81^. While there are seminal studies probing the relative influence of the substrate–liquid and liquid–air interfaces on the quality, quantity, and orientation of interface-nucleated semiconducting polymer crystallites^82,83^, there has been limited research into the control of such nucleation specifically using a solution-phase coating process. While few studies have demonstrated the manipulation of the fluid dynamics during polymer processing to alter nucleation density or out-of-plane crystallite orientation, some have investigated chemical modification or the use of substrate treatments to enhance the growth of either face-on or edge-on crystallites^84^. Although the specific requirements depend on the application of interest, a fundamental understanding of the key processes and deposition variables affecting the control of crystallite nucleation is crucial for the development of effective MGC methods.

### Polymer orientation control

While control of the nucleation of polymer crystallites is indeed crucial for tuning the final morphology of films, direct manipulation of the orientation of an ensemble of polymer chains relative to the carrier transport direction, etc. can impart favorable properties to active layers. Alignment of polymer chains in a particular in-plane direction facilitates charge transport because of faster charge transport along the polymer backbone and can reduce device cross talk by imparting charge transport anisotropy, which is a desirable property for OFET arrays. Work in aligning conjugated polymer thin films often reports both enhanced field-effect mobilities in the alignment direction and charge transport anisotropy^85^.

**Fig. 2:**
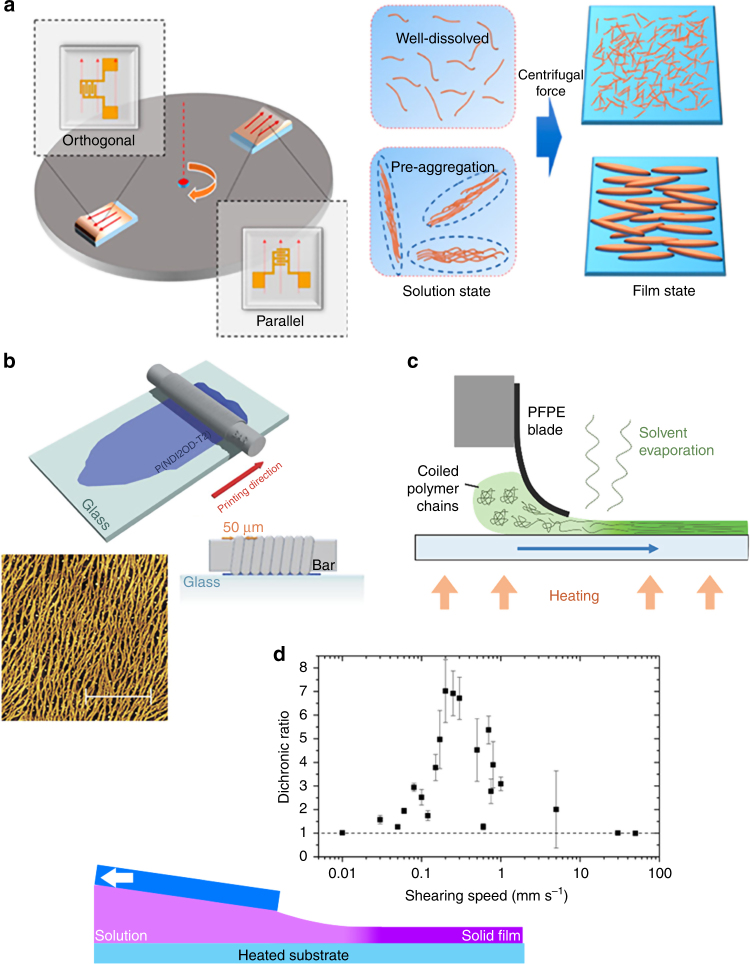
In-plane alignment induced by deposition. **a** Off-center spin coating of only a pre-aggregated polymer solution can induce uniaxial alignment in the resulting film, attesting to the role of aggregates in the in-plane orientation of the dry film. Figure adapted from ref. ^87^ (copyright 2015 American Chemical Society). **b** Bar-coating of naphthalene-dicarboximide-bithiophene polymer solutions yields highly aligned polymer fibers likely because of the high shear strains imposed by the coating bar. Figure adapted from ref. ^85^ (copyright 2015 Nature Publishing Group). **c** Blade coating of a diketopyrryolopyrrole (DPP)-based polymer with a flexible blade similarly results in aligned films because the upper liquid–solid interface provided by the blade induces shear strain greater than what would be possible with a free interface (an air–liquid interface like in dip coating). Figure adapted from ref. ^92^ (copyright 2015 John Wiley and Sons). **d** In another example, the in-plane alignment of DPP-terthiophene polymer thin films as measured by the optical dichroic ratio can be directly tuned by changing the coating speed and thus the effective imposed shear stress. Figure adapted from ref. ^93^ (copyright 2016 American Chemical Society)

MGC methods can be used to create unidirectional gradients and shear stress fields during film deposition in the evaporative regime to cause polymer alignment. Such techniques—which we call “flow-aligning” methods—induce alignment by virtue of the processing method itself—the mechanism of controlling polymer orientation is the direct result of the phenomenon inherent to the process. The directional stress could involve concentration gradients during drying or external forces (mechanical, electric, magnetic, etc.). The generality of these processes can be exploited for different polymer systems with minimal complexity added to the overall processing scheme, and they include zonecasting^86^, off-axis spin coating (Fig. 2a)^87^, directional drying in a capillary^11^, thin-film compression^88^, dip coating^89^, and Langmuir–Blodgett methods^90^ among others (Fig. 2b, c). Maximizing the shear stress in a particular direction should enhance chain alignment in that direction, which for MGC, generally implies faster coating speeds. However, in the LL regime, relaxation of the polymer likely limits any achievable alignment, given that spreading of the wet film and drying are separate processes. In the evaporative regime, the thinner film requires less time for all of the solvent to evaporate, thus causing more rapid supersaturation and increasing the stochastic nucleation of randomly oriented crystallites and reducing overall alignment. In this way, an optimal coating speed balancing these two factors is necessary, but it is difficult to a priori predict the ideal printing conditions for high in-plane alignment of a given polymer. The influence of shear forces in the solution is difficult to directly measure, but fluid dynamical simulations could offer important insights.

While it is unclear whether the alignment of (semicrystalline) conjugated polymers in thin films results from directly shear-aligning individual, dissolved polymer molecules, or larger aggregate/assemblies, it is likely that aggregates whose overall shape is elongated or at least anisotropic can be aligned under shear stress and may play an important role in overall alignment. For MGC involving shear strain, studies often achieve highly aligned, solution-printed polymer thin films by relying on the formation of fibril aggregates in solution. Polymer inks can be treated with ultraviolet (UV) or aged to promote the fibril formation, after which alignment of these crystalline domains is achieved using MGC^91^. In many of these cases, reports in the literature have highlighted systems—solvent, polymer, deposition, temperature, and coating mechanism combinations—where polymer aggregation via the formation of well-defined fiber morphologies is crucial for the realization of alignment (Fig. 2b, c)^85,87,92^. However, recent work has also demonstrated that both the crystalline and amorphous regions of diketopyrryolopyrrole-based donor–acceptor polymer thin films could be uniaxially aligned using MGC, achieving optical dichroic ratios—a metric for in-plane alignment in donor–acceptor polymers—as high as 7 (Fig. 2d)^93^. It is possible that shear stress is effective at aligning these larger aggregate structures as opposed to dissolved free polymer molecules, but the role of crystallites during film formation is unclear. Specifically, it is not known if the presence of aggregates, and thus some degree of crystalline behavior, is essential for effective shear alignment and whether their size scale or shape is important.

We note that reports using flow-aligning methods are much less prevalent than those using pre- or post-deposition processing techniques or those exploiting intrinsic liquid crystallinity. The latter induce alignment in a process step separate from the actual deposition^94^ and include post-deposition mechanical abrasion^95^, mechanical deformation^96^, external fields^97^, and soft lithographic processes^98^. Early work in polymer OSC alignment revolves around the ability of polymers with intrinsic LC phases to template on a topologically ordered substrate that induces preferential in-plane alignment^99^. Ordered surfaces can be fabricated by unidirectional friction transfer to^100^ or abrasion of^101^ a substrate^102^. Conjugated polymer solutions are then cast with^86^ or without^103^ additional heat treatment to assist polymer alignment, which effectively transfer the alignment of the underlying substrate to upper polymer layer.

As a general principle, exploiting a polymer’s intrinsic properties—ordered LC phases, associative interactions, etc.—can yield very high control over a deposited film’s orientation. The key here is the natural proclivity for ordered self-aggregation and the ordered topology of the substrate, which, while restricting this type of multistep alignment to polymers with accessible LC phases, provides a key insight: in integrating both the design and processing of tailored semiconductors, a balance must be struck between high aggregation energy and processability. The former may improve *π*-stacking and the possible formation of alignable, anisotropic crystallites, while the latter generally calls for reduced self-association and the incorporation of solubilizing moieties that are generally non-conductive. There are many questions left unanswered: How important is shear strain for alignment? What is the effect of solvent on the proportion of free, dissolved polymer molecules versus *π*-stacked aggregates? Which components of solid film formation—polymer vitrification, solvent evaporation rate, nucleation density, etc.—influence alignment? How do polymer nucleation and crystallite growth inhibit or facilitate alignment?

As a last point, we would like to mention the relative lack of studies on controlling the out-of-plane orientation of polymer semiconductors using MGC methods. While much work in this space is focused on chemical modification and side chain chemistry to control the surface energy of conjugated polymers (beyond the scope of this review), only a few reports specifically use the method of deposition as the primary mechanism. For example, solution shearing has been shown to influence the out-of-plane orientation of P3HT crystallites for in P3HT/PCBM ([6,6]-phenyl-C_61_-butyric acid methyl ester) blends^104^. The crystallites orient mostly edge-on when coated at low speeds but change to a mixture of edge-on and face-on orientations at high speeds. In another report, polarized soft X-ray scattering reveals that solution shearing induces more face-to-face orientation of isoindigo-based polymers relative to the interface of two components than spin coating, with this effect being more pronounced when a slow shearing speed is used rather than a fast one^105^. For applications, like OPVs, requiring multicomponent systems, the influence of the deposition method is intricately convolved with the thermodynamics and kinetics of phase separation, which must be considered.

### Phase separation for multicomponent films

**Fig. 3:**
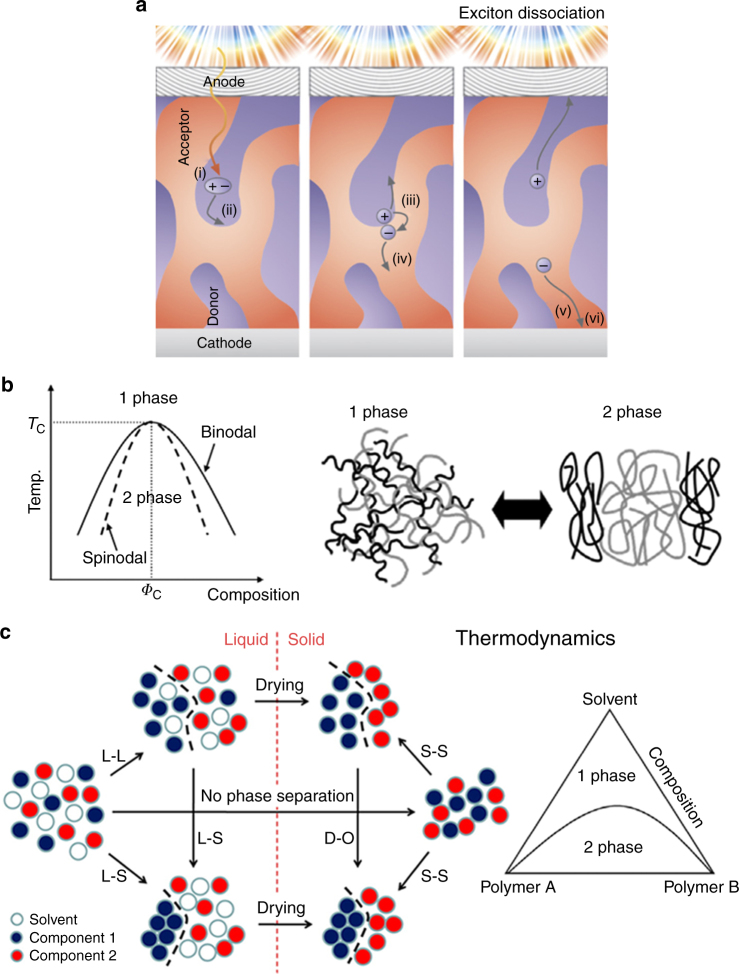
Phase separation of multicomponent systems. **a** Diagram of the OPV operation, which consists of (i) excitation by a photon, (ii) migration of an exciton to a donor–acceptor interface, (iii) exciton dissociation by charge transfer, (iv) charge separation, (v) migration to the electrode, and (vi) charge extraction. One key to effective energy harvesting is the effective dissociation of coulombically bound excitons at the domain boundaries separating the donor and acceptor before the exciton recombines. The exciton diffuses on average about 10 nm before it recombines, thus implying that the optimal domain size should be around the same length scale. The thermodynamic phase diagrams describing a simple two-component polymer system (**b**) and a ternary diagram addressing the role of the solvent (**c**). Multiple processes can occur as the non-equilibrium state of the drying film’s microstructure evolves, and therefore overall film evolution is likely a combination of both kinetically limited and thermodynamically limited processes. Figures adapted from ref. ^108^ (copyright 2010 IOP Science), ref. ^127^ (copyright 2014 MDPI), and ref. ^114^ (copyright 2013 American Chemical Society)

In organic solar cells, the phase-separated domain size is one of the most important morphological characteristics that determines the exciton harvest efficiency and that profoundly affects device performance^1,106,107^. OPVs constitute a very active area of research for an application requiring the interaction of two or more chemically dissimilar components in solution. The resulting dried film active layer is a BHJ composed of donor and acceptor materials, whether they are polymers, small molecules, or any combination thereof. Unlike in inorganic semiconductors, light absorption in OPVs generates coulombically bound excitons rather than free charge carriers due to the lower dielectric constant of organic materials^108^. Charge dissociation, required for subsequent collection at electrodes, necessitates excitons to diffuse to a boundary between donor and acceptor domains, where there is a driving force for exciton dissociation (Fig. 3a)^109^. Domains much larger than the exciton diffusion length (~10 nm) will suffer from exciton recombination, resulting in reduced photocurrent^12^. For this reason, any coating technique that affords a level of control over the BHJ morphology is a critical tool for realizing high-performance OPVs. The key performance metric is the overall PCE under AM 1.5 spectral irradiance, with the current record exceeding 12%^110^ as compared to silicon cells with PCEs of >20%.

Amorphous polymer blends are driven to phase separate by the system’s proclivity to minimize its Gibbs free energy, as described by the classical Flory–Huggins solution theory^69^. According to this model, the interaction between donor and acceptor is described by the Flory–Huggins parameter *χ*, which is a function of temperature and can be estimated from the Hildebrand solubility parameters of the two components^111^. These considerations pertain to the thermodynamic equilibrium state when the free energy is minimized, and thus it is equally important, if not more, to consider the kinetic processes that occur during rapid solvent evaporation. The pairwise diffusion constants (diffusivities) of a given polymer with respect to the solvent and the other components of the system determine its mass transport rate, which can strongly affect phase separation as a consequence^112^. Properties like longer polymer chains and aggregation can cause much slower chain diffusion due to entanglements^113^ and inter-chain interactions. For semicrystalline conjugated polymers, the phase separation process is likely a combination of spinodal decomposition—the spontaneous de-mixing described above—and polymer nucleation and crystallization, which in contrast involves a free energy barrier (nucleation is an exergonic transition only when the system is above the threshold for supersaturation and is otherwise endergonic)^114^. It is unclear what the relative importance of these two processes are in general and whether or not their influence can be rationally controlled—previous publications suggest that phase separation is strongly material-dependent because both phenomenon have been observed^43,49,115,116^. Due to the complexity in simulating both the thermodynamics and kinetics encountered during solid film formation, phase separation has not been precisely predicted despite efforts via computational modeling^117^. Processes like interfacial segregation and vertical film stratification can be controlled with a firm understanding of these fundamental thermodynamic and kinetic processes, and further research could lead to effective methods of driving down domain sizes and of inducing beneficial film morphologies.

**Fig. 4:**
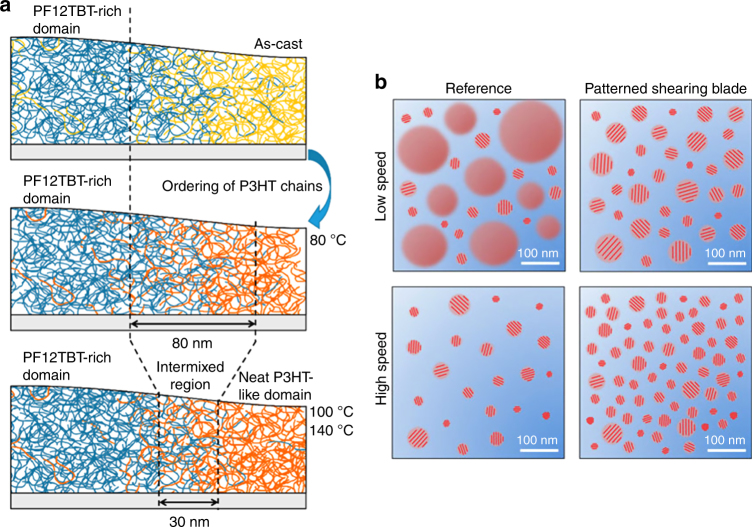
Film evolution of multicomponent systems involving phase separation. **a** The evolution of the interface between two polymer components in a dry film as a function of thermal annealing as determined by their thermodynamic mixing parameters. Increasing the temperature above the glass transition point post deposition reduces the size of the intermixed region separating donor-rich and acceptor-rich domains. Figure adapted from ref. ^112^ (copyright 2017 American Chemical Society). **b** Manipulation of domain size and nucleation density caused by the use of a patterned coating blade to induce extensional flow and increased shear strain. Within the amorphous electron-acceptor polymer matrix (blue), red domains indicate amorphous electron-donor polymer while domains with red bars indicate semicrystalline regions. Higher coating speeds using a blade patterned with pillar structures that causes extensional flow induce the formation of smaller, more numerous nuclei, which become small crystalline domains in the solid film. Figure adapted from ref. ^79^ (copyright 2015 Nature Publishing Group)

Because the BHJ active layer is a kinetically trapped system resulting from the spontaneous phase separation between donor and acceptor polymers during solvent evaporation (Fig. 3b, c), controlling device morphology is particularly challenging. Early studies aiming to scale up OPV fabrication using MGC methods reveal substantial efficiency drops compared to spin-coated films^118,119^. Such a difference in device performance can be attributed to several factors, including differing active layer morphology, substrate roughness, interfacial layers near electrode, processing methods, and device structures, but film morphology is likely the most crucial parameter influencing efficiency as revealed by recent studies^120^. The difficulty in controlling the requisite phase separation has led to an enormous amount of research toward obtaining small domains using common strategies like thermal annealing (Fig. 4a)^112^, the use of processing additives or co-solvents, concentration variation, etc., but surprisingly few studies have explored the role of the deposition technique itself. We find that MGC methods are underutilized in the OPV literature to control morphology despite a profound ability to influence domain size, crystallinity, and alignment in OSC devices. Furthermore, to truly realize the promise of high-throughput production, it is necessary to move beyond spin coating to linear methods like MGC^121^.

Given that cost is an important parameter to consider for industrial production, high throughputs involving high coating speeds (e.g., >1 m min^–1^) are desirable for industrial scale-up^122^. Consequently, most industrial-scale slot die R2R coating processes operate at high speeds well within the LL regime. As discussed previously, rational ink formulation can dramatically influence the final film morphology, but the fluid mechanical effects of the deposition method on phase separation, however, have received far less attention. A few studies demonstrate that differing coating speeds can affect the phase separation between donor and acceptor in the LL regime^123^, but an optimal set of deposition conditions may actually reflect the greater influence of some variables—like dry film thickness—over others^124^. Because factors like the relative speed of solvent evaporation, film thickness, and polymer nucleation rate are interrelated, additional work is need to deconvolve such effects. When the entanglement of polymer chains and the aggregation between different chains dominates, shear strain likely has a limited effect on phase separation: as recently demonstrated, the phase separation of a mixture of high-molecular-weight donor and acceptor polymers is insensitive to coating speed^49^.

On the other hand, the evaporative regime is less explored for OPV deposition. Although the coating speeds involved may not be directly useful for the industrial printing of OPVs, deposition in this regime can reveal methods to tune film morphology by modulating the flow and shear fields in the solution. As mentioned previously, the ink meniscus generally has a short length (<1 cm), and the flow within the meniscus is a delicate balance between mass and heat transport. Because deposition is under the direct influence of the meniscus, the non-equilibrium state of the ensemble of polymer chains (e.g., extended chains, flow-induced nuclei, aligned molecules, etc.) could be “frozen” into the dry film. Shear strain and extensional flow can be used to control phase separation, overall crystallinity, and domain orientation. In one study, a micro-patterned shearing blade rationally designed to locally increase shear strain and induce extensional flow is shown to enhance the crystallinity of one polymer component, causing an improvement in OPV performance and, most importantly, a reduction of the phase separation scale (Fig. 4b)^79^. This work is particularly notable because it is hard to enhance crystallinity without increasing of the phase separation size scale since both are coupled—crystallization is a strong driving force for the system to phase separate. In another study, albeit of polymer/small-molecule blends, higher coating speeds reduce the average phase separation size scale from 100 nm (1.0 mm s^–1^) to 75 nm (2.5 mm s^–1^)^105^. Other deposition parameters like substrate temperature are also important factors that affect the final deposited BHJ film. For example, higher temperatures result in increased phase-separated domain size and reduced polymer crystallinity in isoindigo/fullerene BHJ films^125^. Moreover, external fields (e.g., electrical^126^ and magnetic) or UV light can be used to aid polymer association and aggregation, methods yet to be explored for OPV deposition. While research in this regime may not be immediately transferable to high-speed MGC, they provide novel methods and insights into the control of the morphology of solution-processed OPV active layers.

## Outlook

MGC processes not only provide a method to align semiconducting polymers and control their film morphologies but can also serve as a lab-scale platform for fundamental studies of the complex phenomena that occur during deposition, contributing to a fuller picture of structure-processing-performance relationships and resulting in the steady increase in the performance of OSC devices. Notably, in situ morphology evolution studies during MGC have yielded significant insights into polymer crystallization and the dynamics of film formation^13^. This highlights an important aspect in improving organic semiconductor devices: we emphasize that a polymer’s chemical structure and the manner by which it is processed should not be treated as separate, orthogonal factors, and the study of these factors requires an interdisciplinary approach drawing upon physics, chemistry, and material science. While polymer chemical structure and film processing have been shown to be important individually, considering the important interplay between them will lead to unique approaches for producing the next generation of semiconducting polymer devices, and we anticipate a bright future for solution-processed polymeric OSC devices.

Still, numerous significant challenges remain. Looking forward, we highlight three key research directions that we believe are critical to the continued development of solution-deposited, high-performance OSC thin films.

### Ink formulation studies

The in-depth study of conjugated polymer ink formulation is lacking. A fundamental understanding of OSC polymer chain conformation (coiled, wormlike, and aggregated in solution) would also bring new insights. Concentration effects, non-Newtonian behavior, and other fluid mechanical considerations have not been carefully studied for such solutions of these materials; rheology work along this line would greatly benefit the field. In line with the goal of high-throughput production, the use of non-toxic industrial solvents must be addressed. This is challenging since non-halogenated compounds are typically unsuitable for strongly self-associating polymer OSCs. Co-solvent mixtures or solutions with additives may be employed to enhance dissolution but can also complicate drying dynamics. There are several studies along these lines^116^, but further work on the effects of solvents is warranted with regard to not only evaporation dynamics but also nucleation and crystallization in terms of polymer–solvent, polymer–solvent–nonsolvent, and polymer–polymer–solvent–nonsolvent interactions, which have received very little attention. Lastly, the shelf life of conjugated polymer solutions should be investigated to ensure consistency in device fabrication, given the possibility of aggregation over time.

### Theoretical work

Even though the fundamentals of surface wetting, polymer nucleation and crystal growth, and rheology in the lubrication limit are mature areas of research, there has been little exploitation of this vast corpus of knowledge to the specific application of organic semiconducting polymer active layers—for example, there are many opportunities to extend the knowledge gained from the past six decades of study of the crystallization of polymer molecules to this important area of research. Deposition parameters like coating speed and temperature influence solvent evaporation, polymer nucleation rate, dry film thickness, and diffusion, and the likely nonlinear effect various parameters can have on each of these phenomena must be deconvolved to fully control the deposition process. Furthermore, molecular dynamics simulations and continuum models can specifically benefit the study of the velocity and shear fields during deposition, polymer–polymer phase separation and thermodynamics, diffusion kinetics, and overall microstructural evolution. The need for theorists and computational experts to create new paradigms to approach the issue of polymer nucleation, crystallization, and rheology is significant, and we emphasize that more effort is exerted in the near future toward a more comprehensive understanding of the processes related to the MGC of semiconducting polymers.

### High-throughput and large-area deposition

Studies of research-scale devices, while crucial, often do not take into account the considerations necessary for large-scale production. We emphasize that certain deposition techniques like spin coating are simply not suitable for high-throughput, continuous fabrication. The differing deposition dynamics between various solution processing methods profoundly impact device morphology and performance, and this translational failure from spin coating to something like R2R printing is particularly evident and troubling in OPVs^120^. We believe research efforts should focus on methods that are suitable for the adaptation to large-area production (i.e., linear printing processes), like MGC methods. With this shift in focus, we anticipate new areas of research into the fluid mechanical phenomena inherent to the two-dimensional nature of MGC, which is often simplified with one-dimensional models. Behaviors like viscous fingering and other concentration instabilities may manifest in the direction transverse to the coating direction and may affect the performance of deposited films.

### Box 1 Fluid mechanical phenomena

In the evaporative regime at the forward meniscus downstream of the coating head, the processes underlying meniscus-guided coating are manifold and interrelated. In the lubrication limit (Re « 1), the fluid velocity profile under the coating head is a combination of pressure-driven (parabolic) and boundary-driven (linear) flows denoted by the flow profile on the left. Semiconducting polymer molecules, represented as alternating blue arrows and yellow chevrons, are directly influenced by the resulting shear strain (related to the gradient of the velocity profile), which can both induce uniaxial alignment in the direction of coating and enhance the aggregation and nucleation of the polymer.

As the meniscus passes over the substrate, capillary flow related to the coffee-ring effect brings solution toward the contact line and may be further enhanced with diffusive flow related to the dramatic concentration gradient that arises as the solute concentration increases from that of the solution to the bulk density of the polymer. The forward meniscus itself can be subject to a gradient in surface tension, inducing Marangoni flows at the liquid surface toward the solution bulk (e.g., binary solvent mixtures) or toward the dry film (solutal Marangoni flow). All of these processes occur while solvent evaporation from the air–liquid interface can also cause evaporative cooling at the interface, which may introduce a temperature gradient in the out-of-plane direction to influence each of the previous flows.

The combined effect of each of these phenomena can dramatically alter the final thin-film microstructure of the polymer semiconductor, which includes the nucleation density, crystalline fraction of the film, and the orientation of the crystallites in plane (alignment) and out of plane (e.g., edge-on, face-on, and end-on).
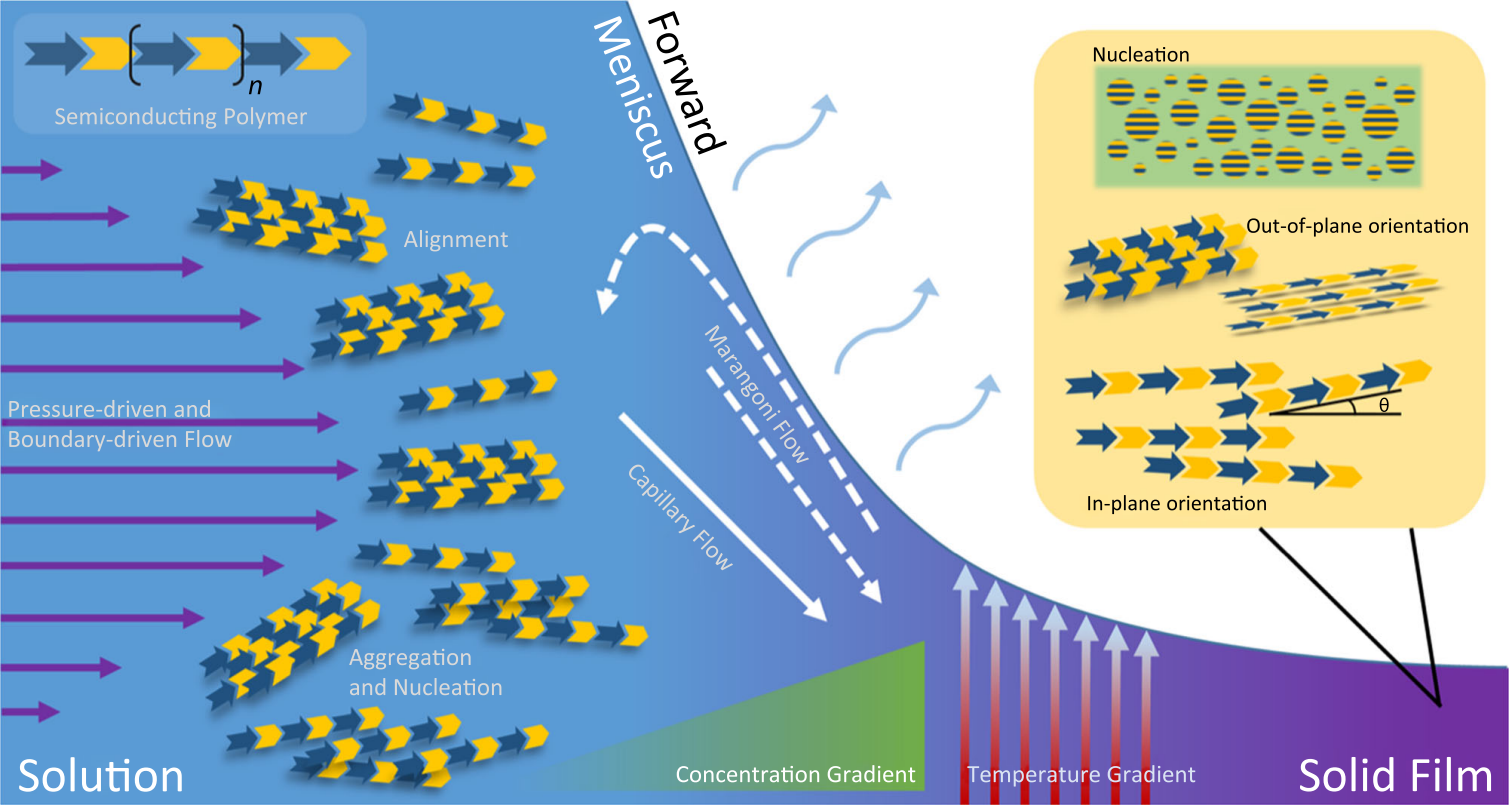


Summary of flows, gradients, and fluid mechanical phenomena that can influence semiconducting polymer molecules and their resulting microstructure in the thin film

### Box 2 Deposition regimes

Two primary deposition regimes related to coating speed typically exist for a combination of deposition parameters (temperature, concentration, solvent, etc.). The evaporative regime occurs at low coating speeds when the characteristic timescales for solvent evaporation and coating velocity are comparable. As a function of coating speed *v*, the regime is characterized by a power-law decrease (exponent: –1) in dry film thickness *t*, and functional dependencies on deposition parameters can be described by a simple mass balance when neglecting possible recirculation flows. At high coating speeds, the classical Landau–Levich regime occurs and is characterized by a power-law increase (exponent: 2/3). Here a wet film is first dragged out before drying, indicating the decoupling of coating and film drying.

The parameters that affect the film thickness in the two regimes are different and reflect what fluid mechanical parameters are involved in the process. In the evaporative regime, evaporated solvent flux *Q*_evap_, solution density *ρ*, solution concentration *c*, and lateral dimension *W* are important. In the Landau–Levich regime, solution viscosity *η*, surface tension *γ*, back meniscus height *l*, blade contact angle *θ*_b_, substrate contact angle *θ*_s_, and capillary length *κ*^–1^ = *γ*/*ρg*^0.5^ are important because the upstream back meniscus plays a role in coating.

In the example of slot die coating, deposition is further complicated by the flow rate of the input ink and has been extensively studied because of its industrial relevance. Flow instabilities can result from unoptimized deposition parameters and lead to poor film morphology. The relevant parameters that affect the minimum wet film thickness *t*_min_ and the zero-pressure-difference film thickness *t*_0_ from the capillary model are the upstream gap height *h*_u_, downstream gap height *h*_d_, meniscus surface tension upstream and downstream *γ*_u_ and *γ*_d_, capillary number Ca, and dynamic contact angle *θ*.
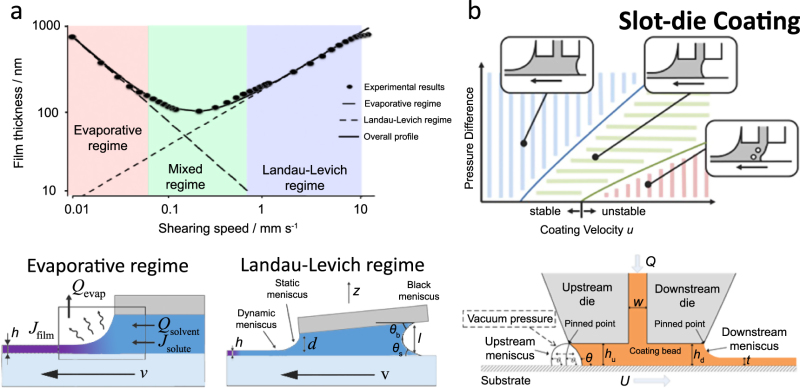


**a** Two deposition regimes characterized by differing thickness behavior as a function of coating speed can exist at deposition temperatures relatively close to the solvent boiling point. The timescale for film solidification decreases as coating speed is increased, transitioning to the classical Landau–Levich regime where a wet film is first dragged out by the blade before the film dries. Figure adapted from ref. ^36^ (copyright 2010 American Chemical Society) and ref. ^35^ (copyright 2009 American Chemical Society). **b** Slot die coating is a pre-metered, industrially relevant MGC method whose deposition parameters must be optimized to prevent coating instabilities. Figure adapted from ref. ^52^ (copyright 2013 Elsevier) and ref. ^50^ (copyright 2016 AIChE)

### Box 3 Nucleation dynamics of polymers

In linear homopolymers like polyethylene, nucleation and crystal growth can be considered a three-part process consisting of polymer aggregation, aggregate extension and coalescence, and continued crystallite growth beyond a critical lamella thickness L*. For simple homopolymers with no additional associative interactions beyond simple dispersive forces, the dissolved, coiled state (in a good solvent) must be deformed into a collapsed, aggregated state, which then extends and coalesces to form stable nuclei that continue to grow by incorporating new polymer molecules. In contrast, (semicrystalline) conjugated polymers often have such strong intra- and intermolecular interactions—vis-à-vis *π*-interactions and (sometimes) hydrogen bonding—that they may already be aggregated in solution during deposition. In such cases, entropic barriers to aggregation during supersaturation are obviated, making the overall crystallization process quite different than for conventional non-conjugated polymers. Thermodynamic treatments of the PE free energy landscape reveal various metastable conformations and can provide insight into the nucleation process and the resulting thin-film microstructure. In contrast, polymer semiconductors likely have an even rougher energy landscape corresponding to a configurational space complicated by intermolecular interactions stronger than the dispersive ones of PE.
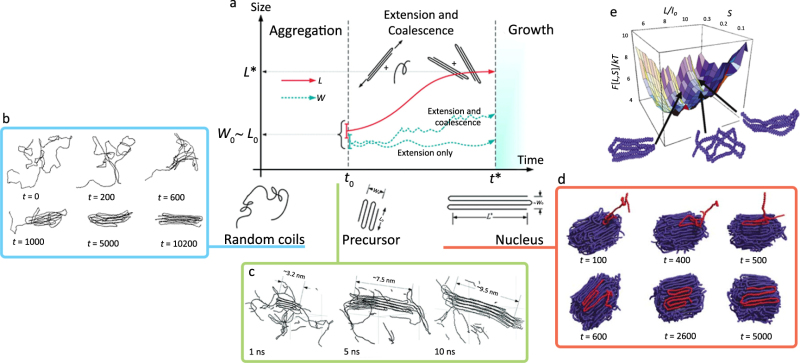


**a** The three phases of linear homopolymer nucleation and crystal growth consist of **b** the aggregation of polymer coils into collapsed structures, **c** the extension of these aggregates with the extension of substituent polymer molecules and the coalescence of other aggregates, and **d** the incorporation of additional polymer molecules into the growing crystallite. **e** The free energy landscape of polyethylene accounts for the various conformational degrees of freedom that can lead to non-equilibrium, metastable thin-film microstructures. Figure adapted from ref. ^65^ (copyright 2010 American Chemical Society), ref. ^64^ (copyright 2000 Elsevier), and ref. ^66^ (copyright 2001 American Physical Society)

## Electronic supplementary material

Description of Additional Supplementary File

Supplementary Data 1
