# Supplementary material for: The meniscus-guided deposition of semiconducting polymers
Source: Nat Commun. 2018 Feb 7;9:534. doi: 10.1038/s41467-018-02833-9 (PMC5803241; doi:10.1038/s41467-018-02833-9)
Supplement: Supplementary file 1 — Description of Additional Supplementary File [file 41467_2018_2833_MOESM1_ESM.pdf]

Description of Additional Supplementary File

File Name: Supplementary Data 1
Description: A supporting table to summarize the device information
